# Changes in Flavor-Related Biomarkers in Pacific Oysters (*Crassostrea gigas*) Following Microplastic Exposure

**DOI:** 10.3390/foods13050765

**Published:** 2024-03-01

**Authors:** Yu Liu, Xiaoyu Teng, Lipin Chen, Shuai Wu, Changhu Xue, Zhaojie Li

**Affiliations:** 1 College of Food Science and Engineering, Ocean University of China, No. 5, Yu Shan Road, Qingdao 266003, China; 21210711063@stu.ouc.edu.cn (Y.L.);; 2 College of Food Science and Technology, Hainan University, Haikou 570228, China

**Keywords:** microplastics, pacific oysters, flavor, amino acids, fatty acids, stress

## Abstract

Microplastics have been an emerging threat to filtering species and the ingestion and impacts of microplastics on oysters are a cause for concern. However, much remains unknown about the effects of microplastics on flavor-related biomarkers in oysters. Herein, a laboratory microplastic exposure with concentrations of 1, 10, and 100 mg/L for 15 days was performed to investigate the impacts of microplastics on the flavor parameters of oysters. Exposure to microplastics changed the odor characteristics of oysters. Microplastic exposure had minor effects on the fatty acid composition; however, significant alterations in free amino acids and nucleotides were observed under the 1 and 10 mg/L exposure groups, respectively. The overall results indicated 10 mg/L of microplastic exposure significantly increased the equivalent umami value of oysters. These findings stressed the effects of microplastics on oysters and would be an important reference for the assessment of the potential risks associated with microplastics in marine edible species.

## 1. Introduction

Marine bivalves are known for their exceptional nutritional value, owing to their chemical composition, among which oysters are recognized as being significant bivalves [1] Globally, oyster is the largest cultured bivalve mollusk, and the world’s capture production of oysters is about 134 tons per year, while its global aquaculture production totaled 643,500 tons in 2020 [2]. Oysters serve as exceptional reservoirs of premium nutrients, encompassing proteins, polysaccharides, docosahexaenoic acid (DHA), eicosapentaenoic acid (EPA), amino acids, and bioactive compounds [3]. Oyster consumption by individuals is extensive due to its popularity and widespread availability as commercial seafood products. In 2017, the global consumption volume of oysters reached approximately 1.0 million tons [4]. Oysters, at the global scale, are principally consumed in their raw form or lightly cooked, ensuring the utmost freshness and exemplary quality [5].

Microplastics (MPs) are defined as plastic particles with sizes smaller than 5 mm [6] and can be classified into primary and secondary MPs. MPs are ubiquitous in various marine media, including offshore, water columns, and deep-sea sediments, with reported concentrations ranging from 1 to 102,000 particles per m^3^ [7,8,9,10]. Oysters, being filter-feeding organisms, obtain their nourishment by ceaselessly filtering suspended particles from seawater via their gills [11]. Oysters may unintentionally or deliberately ingest MPs due to their small size and the resemblance of these particles to their natural food [12]. Based on the findings ofWootton, MPs were detected in oysters worldwide, with a prevalence rate of 94.4% [13]. The ingestion of MPs in oysters can be harmful, and several studies have reported the ingestion of MPs may cause metabolic dysfunction, oxidative stress, growth retardation, and reproductive toxicity. For example, polystyrene (PS) MPs inhibited the amino acid metabolism and accelerated the degradation of amino acids and proteins [14]. Parolini found that PS-MPs modulated the content and composition of fatty acid profiles in Cladoceran [15]. Similarly, Guschina demonstrated that PS-MPs reduced the content of essential fatty acids in freshwater algae [16]. Metabolic dysfunction induced by MP exposure might cause changes in the content of nutrients in oysters, such as essential fatty acids and amino acids, and eventually result in the deterioration in food quality. Despite the extensive investigations, the primary emphasis of research has been placed on examining the biological impacts resulting from the exposure of oysters to MPs. However, there are still significant gaps in understanding of the influence of MP exposure on the taste and overall quality of oysters.

Flavor, which mainly consists of taste and odor, is a crucial aspect to consider in bivalve mollusk production and consumption, as it directly impacts their economic value and consumer choices [17]. Evaluating the flavor characteristics involves considering the physical properties and chemical composition of shellfish and utilizing a set of evaluation biomarkers [18]. Nonvolatile compounds, free amino acids, and nucleotides are key flavor components that affect meat’s taste through synergistic effects [3]. Gas chromatography–ion mobility spectrometry (GC-IMS) is a potent method used to separate and detect volatile organic compounds (VOCs), which offers numerous advantages, including exceptional sensitivity, rapid response time, simplicity in operation, and affordability [19]. GC-IMS has been widely used to analyze and identify the VOCs, including aldehydes, esters, alcohols, and ketones, in marine products, such as shrimp, tuna, and oysters [2,20,21].

In this study, the effects of MP exposure on the flavor characteristics of oysters after exposure to MPs for 15 days were investigated. The volatile flavor differences were performed using GC-IMS. Changes in free amino acids, 5′-nucleotides, and fatty acids were also analyzed and evaluated. Further, the equivalent umami concentration and taste activity value were calculated to better evaluate the changes in the flavor characteristics of oysters exposed to microplastics. To the best of our knowledge, this is the first study to explore the effects of microplastics on the flavor characteristics of *C. gigas*, and would be an important reference for the investigation and assessment of the potential hazards of microplastics in marine edible species.

## 2. Materials and Methods

### 2.1. Sample Collection

Four hundred *Crassostrea gigas* (*C. gigas*) were sampled from an oyster-farming zone located in Qingdao, China (36°16′49″ N, 120°00′36″ E). Intact and live oysters with weights of 90–100 g were washed to remove the external contaminations and microorganisms, and then were transported to the laboratory, where they were acclimated for 3 days. The acclimation conditions were as follows: the salinity was set to 31‰ and the temperature was kept at 15 ± 1 °C by a temperature-regulating device.

### 2.2. Materials and Reagents

Methanol and *n*-hexane were purchased from Sigma-Aldrich Chemical Co. (St. Louis, MO, USA) and sulfosalicylic acid, hydrochloric acid, perchloric acid, and potassium hydroxide were provided by Sinopharm Chemical Reagent Co., Ltd. (Shanghai, China). All other chemical reagents used in this study were analytical grade.

### 2.3. Preparation of MPs

Polystyrene (PS) with sizes ranging from 50 to 100 μm were prepared as mixed micropowders and were purchased from Ruixiang polymer material, Dongwan Guangdong province, China. In order to prevent the clumping and adhesion of the polymers to the tank walls, a small quantity of surfactant Tween 20 (0.00001% *v*/*v*) was incorporated in the formulation of the MP stock suspensions [22]. Following the preparation process, the MP solutions were kept at 4 °C and underwent sonication before utilization.

### 2.4. Exposure Assay

Following the acclimation period, oysters were randomly chosen and conditioned in 4 experimental 100 L tanks per treatment. To evaluate the dose effect of MPs on flavor biomarkers of *C. gigas*, oysters were exposed to 3 amounts of MPs: 1 mg/L, 10 mg/L, and 100 mg/L, respectively. During the exposure period, half of the seawater was renewed daily; subsequently, the MP stock suspensions were added. The experiment lasted for 15 days and oysters were sampled in a random manner from each group, and the tissues were separated from the shell and frozen using liquid nitrogen. Subsequently, the tissues were homogenized into powder and placed at −40 °C until further examination.

### 2.5. Microplastic Ingestion

Digestion of soft tissues was performed by [23], with some modifications. Briefly, 6 oysters were homogenized and 5 g samples were added into a 500 mL glass beaker. Subsequently, 10% (*m*/*v*) KOH was added to each glass beaker to digest the oysters. The containers were covered using aluminum foil to avoid air contamination and then subjected to 60 °C for 48 h in an oven. Upon the absence of any organic remains and the attainment of a transparent and amber solution, the digestion process was considered complete. Subsequently, the digestion solutions underwent filtration under vacuum using 1.0 μm glass microfiber (Whatman GF/B). Each filtering paper was divided into four sections to allow for image capturing under a dissecting microscope (Olpmpus, SZX10, Tokyo, Japan).

### 2.6. Analysis of Fatty Acids

The identification of fatty acids was carried out using the methods of Wang [3]. Crude lipid was methyl-esterified using hydrochloric acid–methanol solutions in a metal bath at 90 °C for 2 h to prepare the fatty acid methyl ester. Subsequently, *n*-hexane was added to the mixture to dissolve the fatty acid methyl ester and an aliquot of an *n*-hexane layer was injected into the injection port of 6980 N gas chromatography (Agilent Technologies, Santa Clara, CA, USA) for analysis. The following parameters were applied: Agilent J&W HP-88, which has a 100 m length, a 0.25 mm ID, and a 0.20 m film thickness; the column’s temperature ranged from 180 °C to 220 °C (3 °C per minute) and was kept for 27 min; nitrogen was the carrier gas. By comparing the retention times to those of standard fatty acid methyl ester mixtures from Sigma (St. Louis, MO, USA), the fatty acids were identified.

### 2.7. Analysis of Free Amino Acids

The examination of the oysters’ free amino acids followed the protocol described by Wang with a few minor adjustments [3]. Initially, 2 g of the samples were homogenized using 10 mL of 0.02 mol L^−1^ hydrochloric acid (Sinopharm Chemical Reagent Co., Ltd., Shanghai, China). The mixture was centrifugated at 5000× *g* and 4 °C for 15 min. This process was performed twice; subsequently, the mixed supernatants were blended with sulfosalicylic acid (Sigma-Aldrich, St. Louis, MO, USA). The mixture underwent centrifugation at 12,000× *g* and 4 °C for 15 min. To ensure purity, the resulting solution was filtered via a filtering membrane before being subjected to analysis using an amino acid analyzer (L-8900, Hitachi, Ltd., Chiyoda-ku, Tokyo, Japan).

### 2.8. Analysis of 5′-Nucleotides

The analysis of the nucleotides was performed according to our previous study [24]. Specimens of oysters (5 g) were treated using 10% perchloric acid. Subsequently, the mixtures were subjected to centrifugation for 15 min (12,000× *g*, 4 °C). This procedure was repeated twice, and the pH value of the mixed supernatants was adjusted utilizing KOH solutions. The resulting solution was diluted to a volume of 30 mL; subsequently, 10 uL samples were examined via the Agilent 1260 high-performance liquid chromatography (Agilent Technologies, Santa Clara, CA, USA). The parameters were set up as follows: a CAPCELLPAK C18 SG column (4.6 mm × 150 mm; Shiseido Co., Ltd., Tokyo, Japan); mobile phase: 20 mmol·L^−1^ phosphoric acid, 20 mmol·L^−1^ citric acid, and 40 mmol·L^−1^ triethylamine; flow rate: 0.8 mL min^−1^; duration: 40 min; column temperature: 40 °C; detector wavelength: 260 nm. The retention time of each unidentified nucleotide was compared to its counterpart in reference standards for identification.

### 2.9. Analysis of Volatile Components

The identification of the VOCs in *C. gigas* was performed using GC-IMS (FlavourSpec^®^, Gesellschaft für Analytische Sensorsysteme mbH, Dortmund, Germany) according to Xiao et al. with minor adjustments [25]. Oyster samples with a weight of 1.5 g were introduced into a 20 mL headspace bottle. The samples were then subjected to incubation for a duration of 10 min at 60 °C, while being agitated at 500 rpm. To transfer the samples from the bottle to the capillary column, purified nitrogen was utilized as the carrier gas. The nitrogen flow rates were programmed as follows: 2 mL/min for a period of 5 min, 100 mL/min for another 5 min, and finally 150 mL/min for a duration of 7 min. The VOCs present in the samples were subjected to ionization and subsequently separated within an ionization chamber of the ion mobility spectrometer (IMS). For the qualitative assessment of each compound, the retention time (Rt) and drift time (Dt) were compared to the NIST 2014 (National Institute of Standards and Technology, Gaithersburg, MD, USA) and IMS database.

### 2.10. Analysis of Taste Activity Value (TAV)

The TAVs were determined by dividing the concentration of the taste components by their respective threshold values [26]. The TAV of each substance was calculated using Equation (1) [25]:

TAV = A/B(1)

A and B represent the absolute level of each umami taste component in mg/100 g and the threshold of each umami taste component in mg/100 g, respectively.

### 2.11. Analysis of Equivalent Umami Concentration

The umami concentration value (EUC, g MSG/100 g) denoted the amount of monosodium glutamate (MSG) that matched the umami strength of the combined effect of umami amino acids and 5′- nucleotides [27] The calculation of the EUC was represented by Equation (2):

EUC (g MSG/100 g) = ∑a_i_b_i_ + 1218(∑a_i_b_i_) (∑a_j_b_j_) (2)

a_i_: concentration of each umami amino acid (g/100 g) (Asp and Glu);

b_i_: umami amino acid freshness coefficient relative to sodium glutamate (MSG) (Asp: 0.077, Glu: 1.0);

a_j_: concentration of each umami 5′-nucleotide (g/100 g) (5′-IMP, 5′-GMP, and 5′-AMP);

b_j_: taste nucleotide freshness coefficient relative to the IMP (5′-IMP: 1, 5′-GMP: 2.3, and 5′-AMP: 0.18).

### 2.12. Statistical Analysis

The experiments were conducted in triplicate, and the data were analyzed using the SPSS 25.0 software (SPSS Inc., Chicago, IL, USA) through a one-way analysis of variance with Duncan’s multiple comparison method. The results were expressed as the mean ± standard deviation (SD). A significance level of *p* < 0.05 indicated a statistically significant difference.

## 3. Results and Discussion

### 3.1. MP Uptake

**Figure 1:**
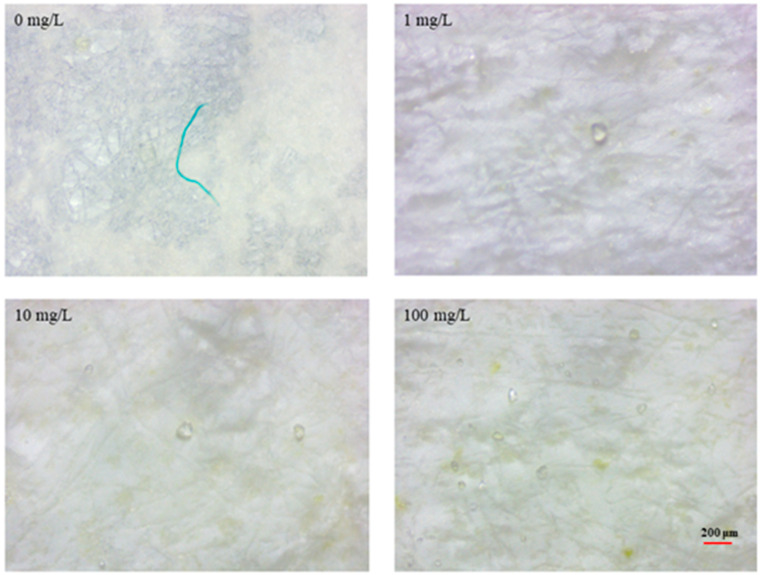
The ingestion of MPs by *C. gigas* under natural and experimental conditions.

At MP exposure, the uptake of MPs was observed under a stereomicroscopy. As shown in Figure 1, with the concentration of the MPs increased, the more MPs were ingested by the oysters. In addition, green fibers were found in the control group, indicating that the oysters could ingest MPs under both natural and experimental conditions. MPs cannot be utilized as energy sources due to the lack of enzymes and enzymatic pathways for their breakdown. MPs ingested by oysters accumulate in the gills and digestive glands of the oysters and induce unfavorable effects in oysters, such as metabolic disorders [22,28].

### 3.2. GC-IMS Analysis

The changes in the VOCs are displayed in Table 1. The IMS drift time and retention index were provided to characterize the VOCs. Since high concentrations of monomer ions and neutral molecules can form dimers in the drift region, a single compound can generate multiple signals, and monomers and dimers of the same compound can also be generated [17]. The monomers and dimers produced were denoted using the letters “M” and “D”, respectively. A total of 85 VOCs were detected, in which 48 VOCs, including 9 alcohols, 17 aldehydes, 13 ketones, 5 esters, 4 ethers, 1 furan, 1 sulfur compound, and 8 other volatile compounds, were identified qualitatively through the NIST database and IMS database, and similar results were also shown in the study of Teng [29].

From the fingerprint plot, it was observed that the experimental groups were richer in VOCs, including alcohols, aldehydes, ketones, and esters, while the control group contained fewer of such VOCs. Alcohols mainly come from the oxidative decomposition of lipids, and the metabolism of amino acids and carbohydrates [30]. Unsaturated alcohols usually have a lower flavor threshold and contribute to the formation of meat flavor, while saturated alcohols have a higher flavor threshold and contribute little to meat flavor [31]. In this study, the key flavor compounds of alcohols in *C. gigas* were 1-pentanol, 3-methyl-1-butanol, 2-methylpropanol, and 1-propanol, and the concentrations of 1-pentanol, 2-methylpropanol, and 1-propanol in *C. gigas* were significantly increased with increasing the doses of MP exposure. This might be MP exposure accelerating the automatic oxidation and degradation of fats.

**Figure 2:**
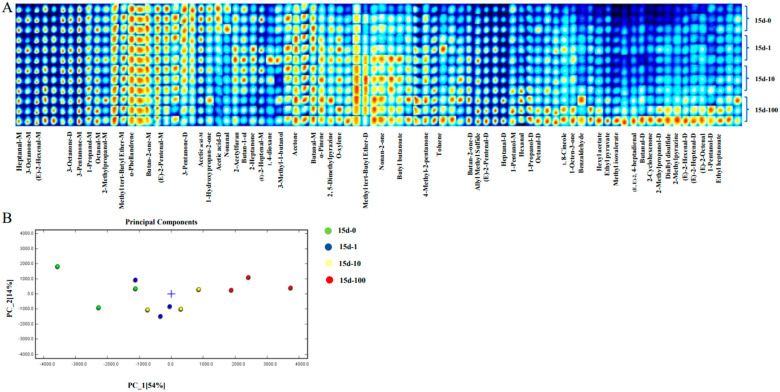
The analysis of volatile compounds of *C. gigas* under MP exposure with different concentrations. (**A**) Fingerprint spectra of VOCs in *C. gigas* based on GC-IMS. The color depth represents the concentration of each component; (**B**) Principal component analysis (PCA) of the VOC profiles of *C. gigas*.

Aldehydes are the main products of lipid oxidation and degradation. Saturated linear aldehydes in marine products are commonly mixed with volatile C8 and C9 compounds and exert a strong effect on the flavor of meat because of their lower flavor threshold, and aldehydes generally have a grassy, fruity, and nutty fragrance at low concentrations, while they may produce an unpleasant fishy smell at high concentrations [32]. As shown in Figure 2, it was found that the concentration of most aldehydes, including Benzaldehyde, (E,E)-2, 4-heptadienal, (E)-2-Octenal, Nonanal, (E)-2-Heptenal, Hexanal, Butanal, (E)-2-Pentenal, and (E)-2-Hexenal, significantly increased in the experimental groups with an increasing MP concentration. Nonanal has aromas of roses and citrus, and hexanal has aromas of grass and cucumber, while Benzaldehyde has a fragrance of bitter almond and (E)-2-Heptenal has a strong oily smell. Owing to the low flavor thresholds of aldehydes, increasing concentrations of such compounds might cause unpleasant smells to *C. gigas.* The production of ketones was related to the thermal oxidation and degradation of unsaturated fatty acids and the protein metabolism [2]. In this study, the levels of most ketones, including 2-cyclohexenone, nonan-2-one, 1-octen-3-one, and 4-Methyl-2-pentanone, in the experimental groups were higher than that in the control group. Although the ketones impart milk and sweet butter flavors, the presence of C7–C11 unsaturated aldehydes and C8 ketones mostly accounted for unpleasant flavors, such as rancid, fusty, oxidized, and musty [33]. 2-Heptatone was mainly produced through linoleic acid oxidation and played a part in modifying the flavor of meat products. Notably, the level of 2-Heptatone was higher in *C. gigas* under MP exposure with concentrations of 1 mg/L and 10 mg/L than that of 100 mg/L, which indicated that a low concentration of MPs induced the increased oxidation of linoleic acid more than a high concentration MPs, leading to the formation of more 2-Heptatone.

It was also found that the concentrations of all esters, including ethyl heptanoate, ethyl pyruvate, butyl butanoate, hexyl acetate, and methyl isovalerate increased with increasing doses of MP exposure. Esters originated from the esterification of alcohols with fatty acids produced by the microbial and enzymatic decomposition of lipids [34,35]. Most low-molecular-weight esters can impart sweet fruity, floral, and wine-like odors. For example, ethyl heptanoate has an aroma of pineapple, butyl butanoate has an aroma of apple, and hexyl acetate and methyl isovalerate have aromas of freshness and sweet fruits. The presence of esters can weaken the pungent taste of fatty acids and the bitter taste of amino groups [36]. According to He et al., the accumulation of esters was related to the increase in bacteria, such as *Candida*, *Bacillus*, and *Aspergillus*, and MPs could provide factitious niches for the colonization of microbes and improve their accumulation in oysters [28,37]. Therefore, it could be speculated that MP exposure caused the accumulation of microbes in oysters, improving the microbial decomposition of lipids and leading to the formation of esters. Other volatile compounds, such as acetic acid and nonanal, which have negative effects on aroma due to their unpleasant smell, were found to have higher concentrations in the control group than in the experimental groups. However, the reasons for changes in such VOCs are unclear and need to be further investigated.

In conclusion, it was found that the odor profile of *C. gigas* could be influenced by MP exposure. Compared with the control group, the concentrations of most aldehydes and ketones, as well as all esters, in the experimental groups were gradually increased with increasing MP exposure doses. The PCA results of the VOCs also showed a separation trend between the control group and experimental groups (Figure 2B), indicating MP exposure affects the VOC profiles of *C. gigas*.

### 3.3. Free Amino Acids Analysis

In marine bivalves, free amino acids serve as vital components for enhancing flavor, and these compounds are essential substrates for body proteins and play a critical role in the regulation of the protein turnover process. In addition, the presence of free amino acids and their catabolites can also function as osmolytes, exerting control over and upholding the intracellular osmotic equilibrium [38]. As shown in Table 2, the profiles of free amino acids in *C. gigas* exposed to MPs were mainly characterized by significantly elevated (*p* < 0.05) levels of branched-chain amino acids (including isoleucine (Ile); leucine (leu), and valine, (Val)), alanine (Ala), histidine (His), and tyrosine (Tyr), together with decreased concentrations of glycine (Gly), threonine (Thr), and serine (Ser), which was similar to the results of Cappello et al. [38].

Aspartic acid (Asp) and glutamic acid (Glu) were MSG-like compounds that imparted aquatic products with an umami flavor [2]. The contents of Asp significantly decreased (*p* < 0.05), while the level of glutamate increased after 1, 10, and 100 mg/L MP treatment compared with the control; however, no significant variance was observed in the total amounts of Glu and Asp. As the precursor of glutathione (GSH), an elevation in glutamate was related to the production of GSH because the toxicity of MPs mainly comes from oxidative stress via the overproduction of reactive oxygen species (ROS) [28], and GSH is an immensely important member of antioxidants, which plays a prominent part in the protective process against oxidative stress. The contents of glycine decreased after the MP treatment, and studies demonstrated that the glycine level is linked to the health status of oysters, observing a decrease in these amino acids after heavy metal and pathogen contamination [22]. Moreover, MP exposure also induced a significant decrease (*p* < 0.05) in threonine, and a similar outcome has been observed in the study of Deng et al. [39]. Threonine is an important antioxidant, and decreased threonine, probably because MP exposure, triggered serious oxidative stress. Therefore, it could be speculated that MP exposure induced oxidative stress and metabolic disorders in *C. gigas*, which could be demonstrated by several studies [22,40,41].

A slight decrease was observed in the contents of sweet amino acids after MP exposure, including serine, threonine, and glycine, and no significant variance was observed in the changes in the total sweet amino acids in the 10 mg/L and 100 mg/L groups. The levels of almost all bitter amino acids, including valine, leucine, methionine, isoleucine, tyrosine, histidine, and lysine, were found to be increased, and a significant increase (*p* < 0.05) was found in the concentration of total bitter amino acids in all experimental groups with respect to the control. The increase in bitter amino acids could mask the umami substances to some extent and probably affected the taste of *C. gigas*.

The total amount of amino acids increased in the three MP groups compared with the control; an especially significant difference was observed in the 1 mg/L MP treatment compared with the control group, which is probably the reason that the catabolism of the proteins may have been elevated. The shortage of energy-supplying substances improved the breakdown of proteins, since the process of protein degradation was accompanied by energy production; Chen et al., 2020 and Lim et al., 2019 [42,43] also found that the total amino acid contents in bronchus epithelial cells were elevated after PS exposure, which was probably because the PS particles’ autophagy process caused protein degradation to use amino acids for cellular fueling.

In conclusion, MP exposure under concentrations of 1, 10, and 100 mg/L resulted in fluctuations in the contents of free amino acids, with the 1 mg/L exposure condition showing the most significant changes. The reason might be ascribed to the MP treatment improving the protein degradation and dysfunction of amino acids, and these changes might eventually influence the taste profiles of *C. gigas* [14].

### 3.4. Fatty Acid Analysis

The changes in the concentrations of fatty acids in the oysters after MP exposure are shown in Table 3. A total of 15 fatty acids, including 5 saturated ones, 3 monounsaturated ones, and 7 polyunsaturated ones, were identified, among which palmitic acid (C16:0) was the major fatty acid, followed by C22:6n-3 (DHA) and C20:5n-3 (EPA), respectively, in all treated and control oysters, and similar results were also shown in the study of Wang et al. [3].

In terms of the effects of the MP concentrations on the composition of the fatty acids of *C. gigas*, there were significant variances in the main fatty acids of *C. gigas* treated with MPs under different concentrations for 15 d, such as C16:0, arachidonic acid (C20:4n-6), EPA, and DHA, among the experimental and control groups. Several studies on oysters have shown that exposure to high concentrations of MPs induced oxidative stress, which could activate the antioxidant defenses to combat the excessive production of ROS, and thereby uphold the cellular redox equilibrium [44]. The implementation of antioxidant defenses requires substantial extra energy. However, potential food shortage and MP retention in the digestive tract of *C. gigas* might increase satiety and disturb feeding behavior, which would lead to reductions in energy reserves; therefore, FAs might be used and reallocated to support defense mechanisms and individual maintenance under MP stress [15,28]. The analysis of the alteration of the FA profile allowed us to understand changes in FAs and identify potentially improved metabolic pathways [15]. In this study, it was found that the abundance of polyunsaturated fatty acids (PUFAs), including EPA, C22:2, and DHA, decreased, while monounsaturated fatty acids (MUFAs), including C16:1, C17:1, and C18:1, increased in MP-treated oysters compared to the control group. A similar modulation trend of MUFAs and PUFAs was also observed in the study of Parolini et al., which found the relative contents of MUFAs were significantly increased while PUFAs decreased in Cladoceran *Daphnia magna* exposed to PS-MPs [15]. The reason might be ascribed to the synthesis of FAs in *C. gigas* towards shorter and monounsaturated fatty acids under high concentrations of MPs. Although the abundance of MUFAs and PUFAs in the experimental groups were changed, no significant variance was observed, and this might be because the sizes of the MPs used in this study were larger. Generally, the ingestion of MPs by oysters is accompanied by their egestion. Previous studies showed that smaller particles exhibit greater bioavailability and necessitate extended egestion time compared to their larger counterparts. For example, Graham et al. found that most of the particles were egested while some of the smaller particles remained within the oysters [45]. Therefore, it could be speculated that the larger MPs used in this study resulted in a shorter retention time and less bioaccumulation in the digestive tract of *C. gigas*, thus minimizing the effects of the MPs on the FA profiles of *C. gigas*.

From the perspective of the effect of MP exposure on the biosynthesis of essential fatty acids, it was observed that the lowest concentration of MPs (1 mg/L) reduced the levels of C18:2n-6 (from 1.84 to 1.64) and C18:3n-3. Similarly, Guschina et al. observed that MP exposure led to a reduction in the relative amounts of C18:2n-6 and C18:3n-3 alongside an increase in C16:0 and C18:1 in algae [16]. That is probably because of the synthesis of C16:0 and C18:1 at the expense of these two essential FAs under MP exposure. However, the high concentrations of particles (10 mg/L and 100 mg/L) increased the levels of C18:2n-6 and C18:3n-3 compared to the control group. Similarly, Romano and Fischer observed an increase in the abundance of C18:2n-6 and C18:3n-3 in black soldier flies after exposure to 50 μm PP particles for 2 weeks [46]. The phenomenon might come from individuals looking to experience a kind of adaptation that tackles the environmental stress, and thus fatty acid homeostasis was re-established under exposure to high concentrations of MPs [15].

Previous studies have shown that MP exposure affected the lipid and fatty acid metabolism in many organisms, including medaka, zebrafish, rainbow trout, and mice [47,48,49,50]. This might be ascribed to MP exposure modulating the activity of various enzymes responsible for the metabolism, such as catalase, uricase, and fatty acid synthase [49]. It was also observed that MPs modulated the levels of a portion of fatty acids in *C. gigas*; however, no significant correlation between MP doses and changes in fatty acid profiles induced by MP exposure was observed. These findings raise questions concerning the impacts of MPs under different types, sizes, and concentrations on the fatty acid profiles of oysters, thus requiring further investigations.

### 3.5. 5′-Nucleotide Analysis

Adenosine monophosphate (AMP), inosine monophosphate (IMP), and guanosine monophosphate (GMP) were the main umami taste substances in oysters. AMP could promote sustainable, complex, and umami taste as well as sweetness, and inhibited bitterness. IMP could impart an umami taste, and its flavor could be strengthened by Glu and some sweet amino acids [18]. The concentrations of 5′-nucleotides were measured in *C. gigas* exposed to MPs under different concentrations. As shown in Table 2, AMP was the main nucleotide component, followed by Hx, GMP, and IMP, in all treated oysters and control oysters. It was found that MP exposure under 1 mg/mL and 100 mg/mL significantly decreased the contents of IMP, while 10 mg/mL significantly increased the contents of IMP compared to the control group. Hx is a bitter nucleotide and GMP could enhance the umami taste. The 10 mg/L MP exposure significantly increased (*p* < 0.05) the contents of GMP and Hx; however, there were no significant changes in the concentrations of Hx and GMP in the oysters exposed to MPs with concentrations of 1 mg/L and 100 mg/L compared to the control group. In addition, it was found that the levels of AMP were higher in all treated oysters compared to the control group, and significant variance (*p* < 0.05) was observed in the 10 mg/L group. Previous studies demonstrated that MP exposure disrupted the feeding activity of oysters and inhibited energy reserves [22]. This might be because the oysters resisted the hostile environment by reducing growth and increasing metabolic demands to maintain normal functions. The main degradation pathway of ATP in *C. gigas* was ATP → ADP → AMP → IMP → HxR → Hx [2]. Therefore, it could be concluded that MP exposure improved the degradation of ATP, leading to increased relative contents of AMP, which could be demonstrated by the significant decrease in the contents of ATP in the 10 mg/L group (*p* < 0.05). Moreover, it was surprising to find that 10 mg/L of MP exposure induced sharp alterations in the contents of 5′ -nucleotides in *C. gigas* compared with the 1 mg/L and 100 mg/L exposure groups. In conclusion, MP exposure induced changes in the levels of 5′ -nucleotides in *C. gigas* despite no clear dose-dependent effect being observed, and this alteration might eventually affect the flavor through synergistic effects with other taste components, such as free amino acids.

### 3.6. TAV Analysis

The determination of the human perception of food flavor relies on the taste threshold of individuals and the concentration of flavor compounds present in the food [3]. The TAV was utilized to examine the taste intensity of compounds to the overall flavor. Compounds yielding a TAV > 1 were considered active in the food flavor analysis, indicating that this compound made a greater contribution to food flavor. High TAV values for Glu, Ala, and His are shown in Table 2. Free amino acids contributed directly to the food taste, and it was found that the TAV of Glu was the highest, indicating that Glu was crucial to the umami taste of oysters, which was consistent with the similar results in *Crassostrea hongkongensis* [26]. The TAV of bitter amino acid, His, was increased, with a significant increase (*p* < 0.05) being observed in all exposure groups. Although bitterness with relatively high levels could mask the umami taste, it could enhance the umami and sweetness of other amino acids under a TAV of less than 1 [3]. The TAVs of 5′-nucleotides are listed in Table 2, and it was found that GMP had the highest TAV compared to the other nucleotides, indicating the better taste provided by this component. No significant increase or decrease in the TAVs of 5′-nucleotides was observed after MP exposure, except for the 10 mg/L experimental group. Despite having a TAV value of less than 1 for all nucleotides, these constituents could potentially enhance the taste of C. gigas by synergistically interacting with other compounds, including free amino acids.

### 3.7. EUC Analysis

The nucleotides that serve as umami-flavoring substances are mainly IMP, GMP, and AMP, which play a crucial role in the characteristic taste of marine products [51]. As umami amino acids, Asp and Glu can impart aquatic products with an umami taste. Therefore, the EUC value was utilized to evaluate the synergistic effect between free amino acids and 5′-nucleotides.

**Figure 3:**
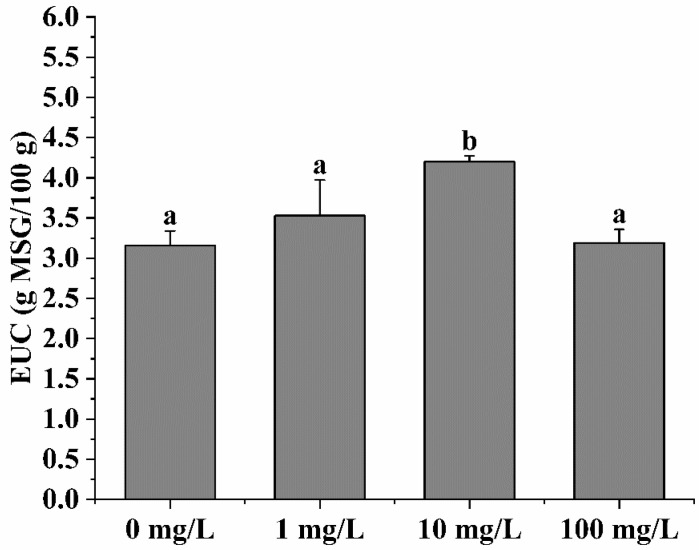
The EUC of *C. gigas* under MP exposure with different concentrations. Bars with different letters were significantly different (*p* < 0.05).

Figure 3 shows that the EUC values were increased after MP exposure, with significant variance (*p* < 0.05) being observed in the 10 mg/L group. Although the amino acid level was not the highest in the 10 mg/L group, the result showed that the EUC of the 10 mg/L group was the highest due to the difference in the relative freshness coefficient. The contents of umami amino acids, specifically Asp and Glu, were found to be connected to the levels of energy compounds like ATP and AMP. Additionally, the EUC value exhibited a positive correlation with the energy level [2]. It was found that the contents of umami amino acids and AMP were highest in the 10 mg/L exposure group compared to the other groups, indicating that the energy levels of *C. gigas* were relatively higher compared to the 1 and 10 mg/L exposure groups.

## 4. Conclusions

This study investigated the impacts of MP ingestion on the flavor-related indicators in *C. gigas*. MP enrichment increased the concentrations of unacceptable volatile compounds in oysters, such as C7–C11 unsaturated aldehydes and C8 ketones. It was found that the 1 mg/L MP exposure significantly influenced the composition of free amino acids, including increased total amounts of amino acids and bitter amino acids compared to the control group, and it was speculated that MP exposure accelerated protein degradation and induced amino acid metabolism disorder. The contents of Glu increased while the levels of Asp decreased after MP exposure, and the changes in the contents of umami acids influenced the taste of the aquatic products. The 10 mg/L MP exposure significantly accelerated the process of ATP degradation, and thus increased the levels of GMP and IMP. The EUC results showed that MP exposure improved the freshness of *C. gigas*, with the 10 mg/L exposure showing a significant difference. Although MP exposure increased the umami taste of *C. gigas*, these alterations induced by MP stress, as well as MPs accumulated in food, might reduce consumers’ purchasing desire and acceptance.

## Figures and Tables

**Table 1:** GC-IMS integration parameters of the VOCs in *C. gigas* under MP exposure with different concentrations.

Nos.	Compound	CAS#	Formula	Molecular Mass	Retention Index	Retention Time	Drift Time
	**Alcohols**						
1	1-Pentanol-M	C71410	C_5_H_12_O	88.1	1253.2	409.705	1.256
2	3-Methyl-1-butanol	C123513	C_5_H_12_O	88.1	1205	349.006	1.2375
3	1-Pentanol-D	C71410	C_5_H_12_O	88.1	1252.5	408.722	1.5108
4	α-Phellandrene	C99832	C_10_H_16_	136.2	1159.4	293.868	1.2288
5	Butan-1-ol	C71363	C_4_H_10_O	74.1	1144.6	277.239	1.1819
6	2-Methylpropanol-M	C78831	C_4_H_10_O	74.1	1096.7	229.519	1.1704
7	2-Methylpropanol-D	C78831	C_4_H_10_O	74.1	1112.1	243.873	1.3626
8	1-Propanol-M	C71238	C_3_H_8_O	60.1	1040.1	187.787	1.1127
9	1-Propanol-D	C71238	C_3_H_8_O	60.1	1038.4	186.699	1.2527
	**Aldehyde**						
1	Benzaldehyde	C100527	C_7_H_6_O	106.1	1497.8	877.504	1.1567
2	(E,E)-2,4-heptadienal	C4313035	C_7_H_10_O	110.2	1479.9	830.551	1.1947
3	(E)-2-Octenal	C2548870	C_8_H_14_O	126.2	1428.2	708.634	1.3319
4	Nonanal	C124196	C_9_H_18_O	142.2	1403.2	656.39	1.4744
5	(E)-2-Heptenal-M	C18829555	C_7_H_12_O	112.2	1333.5	529.882	1.2565
6	(E)-2-Heptenal-D	C18829555	C_7_H_12_O	112.2	1336.3	534.493	1.6777
7	Octanal-M	C124130	C_8_H_16_O	128.2	1293.7	468.738	1.4025
8	(E)-2-Hexenal-M	C6728263	C_6_H_10_O	98.1	1222.6	369.947	1.1841
9	Octanal-D	C124130	C_8_H_16_O	128.2	1292.9	467.501	1.823
10	Heptanal-M	C111717	C_7_H_14_O	114.2	1184.6	324.566	1.3349
11	Heptanal-D	C111717	C_7_H_14_O	114.2	1183	322.588	1.6957
12	Hexanal	C66251	C_6_H_12_O	100.2	1090.2	224.288	1.2643
13	Butanal-D	C123728	C_4_H_8_O	72.1	877.1	108.926	1.2807
14	Butanal-M	C123728	C_4_H_8_O	72.1	875.7	108.417	1.1177
15	(E)-2-Pentenal-M	C1576870	C_5_H_8_O	84.1	1135.1	267.009	1.1063
16	(E)-2-Pentenal-D	C1576870	C_5_H_8_O	84.1	1134.5	266.387	1.3595
17	(E)-2-Hexenal-D	C6728263	C_6_H_10_O	98.1	1217.5	363.825	1.5156
13	Butanal-D	C123728	C_4_H_8_O	72.1	877.1	108.926	1.2807
14	Butanal-M	C123728	C_4_H_8_O	72.1	875.7	108.417	1.1177
15	(E)-2-Pentenal-M	C1576870	C_5_H_8_O	84.1	1135.1	267.009	1.1063
16	(E)-2-Pentenal-D	C1576870	C_5_H_8_O	84.1	1134.5	266.387	1.3595
17	(E)-2-Hexenal-D	C6728263	C_6_H_10_O	98.1	1217.5	363.825	1.5156
	**Ketones**						
1	2-Cyclohexenone	C930687	C_6_H_8_O	96.1	1409.1	668.279	1.1188
2	Nonan-2-one	C821556	C_9_H_18_O	142.2	1396.8	643.522	1.4082
3	1-Octen-3-one	C4312996	C_8_H_14_O	126.2	1308.2	490.282	1.271
4	1-Hydroxypropan-2-one	C116096	C_3_H_6_O_2_	74.1	1309.3	491.943	1.05
5	3-Octanone-M	C106683	C_8_H_16_O	128.2	1263.6	424.036	1.3069
6	3-Octanone-D	C106683	C_8_H_16_O	128.2	1257.7	415.832	1.7175
7	2-Heptanone	C110430	C_7_H_14_O	114.2	1176	313.786	1.2649
8	4-Methyl-2-pentanone	C108101	C_6_H_12_O	100.2	1013.9	171.193	1.1731
9	Butan-2-one-M	C78933	C_4_H_8_O	72.1	903.1	118.584	1.0615
10	Butan-2-one-D	C78933	C_4_H_8_O	72.1	904.4	119.092	1.2473
11	Acetone	C67641	C_3_H_6_O	58.1	816.3	89.313	1.1177
12	3-Pentanone-M	C96220	C_5_H_10_O	86.1	975	149.985	1.1129
13	3-Pentanone-D	C96220	C_5_H_10_O	86.1	986.4	155.675	1.3589
	**Ester**						
1	Ethyl heptanoate	C106309	C_9_H_18_O_2_	158.2	1344.7	548.326	1.4087
2	Ethyl pyruvate	C617356	C_5_H_8_O_3_	116.1	1243.4	396.515	1.1481
3	Butyl butanoate	C109217	C_8_H_16_O_2_	144.2	1235.6	386.413	1.3293
4	Hexyl acetate	C142927	C_8_H_16_O_2_	144.2	1220.3	367.232	1.4061
5	Methyl isovalerate	C556241	C_6_H_12_O_2_	116.2	1049.6	194.25	1.2024
	**Ethers**						
1	1,8-Cineole	C470826	C_10_H_18_O	154.3	1221.3	368.362	1.2974
2	Allyl Methyl Sulfide	C10152768	C_4_H_8_S	88.2	945.9	136.375	1.0428
3	Methyl tert-Butyl Ether-M	C1634044	C_5_H_12_O	88.1	650.2	51.902	1.1222
4	Methyl tert-Butyl Ether-D	C1634044	C_5_H_12_O	88.1	685.4	58.217	1.3516
	**Furans**						
1	2-Acetylfuran	C1192627	C_6_H_6_O_2_	110.1	1489.8	856.337	1.1122
	Sulfur compound						
1	Diallyl disulfide	C2179579	C_6_H_10_S_2_	146.3	1461.4	784.72	1.2016
	**Other**						
1	Acetic acid-M	C64197	C_2_H_4_O_2_	60.1	1467.4	799.316	1.0539
2	2-Methylpyrazine	C109080	C_5_H_6_N_2_	94.1	1294.3	469.671	1.0772
3	1,4-dioxane	C123911	C_4_H_8_O_2_	88.1	1097.3	229.992	1.1151
4	α-Pinene	C80568	C_10_H_16_	136.2	1000.6	163.316	1.2139
5	Acetic acid-D	C64197	C_2_H_4_O_2_	60.1	1466.1	796.146	1.1558
6	2,5-Dimethylpyrazine	C123320	C_6_H_8_N_2_	108.1	1371.4	595.304	1.1083
7	O-xylene	C95476	C_8_H_10_	106.2	1224.6	372.496	1.0631
8	Toluene	C108883	C_7_H_8_	92.1	1050.1	194.615	1.0358

**Table 2:** The taste attributes (+ pleasant, − unpleasant), contents, taste thresholds, and TAVs of free amino acids and 5′- nucleotides (mg/100 g) of *C. gigas* under MP exposure with different concentrations.

	Taste Attributes	0 mg/L	1 mg/L	10 mg/L	100 mg/L	Taste Threshold	TAVs			
							0 mg/L	1 mg/L	10 mg/L	100 mg/L
Free amino acids										
Tau	-	506.02 ± 30.82 ^a^	506.69 ± 0.11 ^a^	513.50 ± 8.41 ^a^	525.81 ± 32.76 ^a^	-	-	-	-	-
Asp	Fresh/sweet (+)	57.15 ± 2.22 ^b^	45.53 ± 0.38 ^a^	45.87 ± 2.04 ^a^	47.37 ± 3.95 ^a^	100	0.57	0.46	0.46	0.47
Glu	Fresh (+)	127.62 ± 4.57 ^a^	142.23 ± 19.92 ^ab^	148.29 ± 2.00 ^b^	129.85 ± 1.62 ^ab^	30	4.25	4.74	4.94	4.33
Ser	Sweet (+)	30.60 ± 4.44 ^b^	27.44 ± 8.06 ^ab^	19.50 ± 0.40 ^a^	25.17 ± 0.95 ^ab^	150	0.20	0.18	0.13	0.17
Thr	Sweet (+)	18.24 ± 6.09 ^b^	15.00 ± 0.78 ^ab^	9.00 ± 0.76 ^a^	10.02 ± 1.03 ^a^	260	0.07	0.06	0.03	0.04
Gly	Sweet (+)	97.24 ± 44.05 ^a^	77.65 ± 6.07 ^a^	71.92 ± 4.46 ^a^	64.70 ± 7.76 ^a^	130	0.75	0.60	0.55	0.50
Ala	Sweet (+)	113.53 ± 6.71 ^a^	178.22 ± 2.76 ^c^	140.73 ± 3.91 ^b^	146.30 ± 12.71 ^b^	60	1.89	2.97	2.35	2.44
Arg	Sweet/bitter (+)	30.58 ± 0.97 ^a^	47.45 ± 1.51 ^d^	36.77 ± 2.02 ^b^	43.41 ± 2.56 ^c^	50	0.61	0.95	0.74	0.87
Val	Sweet/bitter (−)	4.09 ± 0.41 ^a^	4.89 ± 1.02 ^a^	4.42 ± 0.36 ^a^	4.64 ± 0.32 ^a^	40	0.10	0.12	0.11	0.12
Leu	Bitter (−)	4.90 ± 1.24 ^a^	7.75 ± 0.20 ^b^	5.49 ± 0.30 ^a^	5.82 ± 0.03 ^a^	190	0.03	0.04	0.03	0.03
Met	Bitter/sweet (−)	4.90 ± 1.75 ^a^	8.31 ± 1.28 ^b^	6.80 ± 0.17 ^ab^	8.09 ± 0.39 ^b^	30	0.16	0.28	0.23	0.27
Ile	Bitter (−)	3.32 ± 1.82 ^a^	5.64 ± 0.80 ^b^	5.18 ± 0.02 ^ab^	5.23 ± 0.28 ^ab^	90	0.04	0.06	0.06	0.06
Tyr	Bitter (−)	6.60 ± 0.66 ^a^	10.18 ± 1.11 ^b^	5.53 ± 0.90 ^a^	5.81 ± 0.40 ^a^	-	-	-	-	-
His	Bitter (−)	5.00 ± 1.08 ^a^	8.61 ± 0.76 ^b^	7.62 ± 0.96 ^b^	11.29 ± 0.40 ^d^	20	0.25	0.43	0.38	0.56
Pro	Sweet/bitter (+)	107.02 ± 6.65 ^a^	154.04 ± 15.48 ^d^	127.25 ± 5.80 ^c^	116.38 ± 0.70 ^bc^	300	0.36	0.51	0.42	0.39
Lys	Sweet/bitter (−)	13.71 ± 1.77 ^a^	15.28 ± 1.44 ^a^	15.31 ± 0.37 ^a^	9.06 ± 0.79 ^b^	50	0.27	0.31	0.31	0.18
UFFAs	-	184.78 ± 6.80 ^a^	187.75 ± 19.73 ^a^	194.16 ± 3.85 ^a^	177.22 ± 2.33 ^a^	-	-	-	-	-
SFFAs	-	397.22 ± 48.34 ^a^	499.80 ± 13.78 ^b^	405.14 ± 2.83 ^a^	405.98 ± 9.74 ^a^	-	-	-	-	-
BFFAs	-	42.49 ± 2.61 ^a^	60.65 ± 4.70 ^d^	50.34 ± 1.93 ^b^	49.94 ± 0.86 ^b^	-	-	-	-	-
EFFAs	-	49.14 ± 5.29 ^a^	56.85 ± 4.18 ^b^	46.17 ± 0.57 ^a^	42.86 ± 1.30 ^a^	-	-	-	-	-
TFFAs	-	1270.40 ± 70.80 ^a^	1473.82 ± 29.31 ^b^	1325.99 ± 9.54 ^a^	1328.50 ± 50.65 ^a^	-	-	-	-	-
Nucleotides										
ATP	-	89.95 ± 4.09 ^b^	75.18 ± 3.90 ^a^	72.67 ± 0.18 ^a^	77.03 ± 0.24 ^a^	-	-	-	-	-
Hx	-	7.42 ± 0.26 ^a^	7.35 ± 0.05 ^a^	7.95 ± 0.00 ^b^	7.28 ± 0.27 ^a^	-	-	-	-	-
GMP	-	6.37 ± 0.22 ^a^	6.32 ± 0.04 ^a^	6.82 ± 0.00 ^b^	6.26 ± 0.22 ^a^	12.5	0.51	0.51	0.55	0.50
IMP	-	2.02 ± 0.07 ^b^	1.88 ± 0.00 ^a^	2.44 ± 0.09 ^c^	1.77 ± 0.05 ^a^	25	0.08	0.08	0.10	0.07
AMP	-	12.10 ± 1.99 ^a^	14.95 ± 1.17 ^b^	20.87 ± 0.39 ^b^	14.75 ± 1.75 ^a^	50	0.24	0.30	0.42	0.30

Data are mean ± standard deviation (*n* = 3). Different letters within a row indicate a significant difference (*p* < 0.05). Tau, taurine; Asp, aspartic acid; Glu, glutamic acid; Ser, serine; Thr, threonine; Gly, glycine; Ala, alanine; Val, valine; Pro, proline; Lys, lysine; Ile, isoleucine; Leu, leucine; His, histidine; Arg, arginine; Met, methionine; Tyr, tyrosine; UFFAs, the total amounts of Asp and Glu; SFFAs, total amount of sweet free amino acids, including Thr, Ser, Gly, Ala, Arg, and Pro; BFFAs, total amount of bitter amino acids, including Val, Met, Ile, Leu, Tyr, Lys, and His; EFFAs, total amounts of essential amino acids, including Thr, Val, Leu, Met, Ile, and Lys; TFFA, total amounts of free amino acids; ATP, adenosine triphosphate; Hx, hypoxanthine; AMP, adenosine monophosphate; IMP, inosine monophosphate; GMP, guanosine monophosphate.

**Table 3:** Fatty acid profiles (% of total fatty acids) of oysters under MP exposure with different concentrations.

Fatty Acids	0 mg/L	1 mg/L	10 mg/L	100 mg/L
C14:0	1.24 ± 0.13 ^a^	1.78 ± 0.53 ^a^	1.55 ± 0.15 ^a^	1.28 ± 0.25 ^a^
C15:0	0.27 ± 0.09 ^a^	0.15 ± 0.06 ^a^	0.32 ± 0.15 ^a^	0.33 ± 0.20 ^a^
C16:0	20.14 ± 1.08 ^ab^	21.37 ± 0.83 ^b^	19.59 ± 0.35 ^a^	19.96 ± 1.01 ^ab^
C16:1	1.33 ± 0.12 ^a^	1.38 ± 0.74 ^a^	1.27 ± 0.12 ^a^	2.46 ± 1.36 ^a^
C17:0	0.60 ± 0.04 ^a^	0.60 ± 0.10 ^a^	1.07 ± 0.27 ^b^	0.72 ± 0.06 ^a^
C17:1	1.75 ± 0.33 ^a^	1.73 ± 0.27 ^a^	2.00 ± 0.12 ^a^	1.81 ± 0.16 ^a^
C18:0	5.09 ± 0.32 ^a^	4.73 ± 0.18 ^a^	4.96 ± 0.30 ^a^	5.10 ± 0.25 ^a^
C18:1	6.41 ± 0.85 ^a^	7.42 ± 1.37 ^a^	6.44 ± 1.14 ^a^	6.53 ± 0.94 ^a^
C18:2n-6	1.95 ± 0.24 ^ab^	1.44 ± 0.41 ^a^	2.13 ± 0.35 ^b^	1.84 ± 0.17 ^ab^
C18:3n-6	2.68 ± 0.80 ^a^	1.86 ± 0.20 ^a^	2.69 ± 0.27 ^a^	2.50 ± 0.16 ^a^
C18:3n-3	2.66 ± 1.04 ^a^	3.09 ± 0.26 ^a^	3.40 ± 1.07 ^a^	3.80 ± 0.52 ^a^
C20:4n-6	3.51 ± 0.35 ^a^	3.17 ± 0.34 ^a^	3.45 ± 0.33 ^a^	3.74 ± 0.22 ^a^
EPA	7.21 ± 0.17 ^ab^	8.48 ± 1.32 ^b^	6.67 ± 0.73 ^a^	6.46 ± 0.13 ^a^
C22:2	5.02 ± 0.14 ^a^	4.30 ± 0.83 ^a^	4.82 ± 0.37 ^a^	5.45 ± 0.88 ^a^
DHA	12.57 ± 0.83 ^a^	13.65 ± 1.37 ^a^	12.36 ± 0.46 ^a^	11.22 ± 1.99 ^a^
SFA	27.35 ± 1.52 ^a^	28.64 ± 1.03 ^a^	27.51 ± 0.73 ^a^	27.41 ± 1.58 ^a^
PUFA	35.62 ± 1.91 ^a^	36.01 ± 1.55 ^a^	35.54 ± 0.54 ^a^	36.66 ± 1.53 ^a^
UFA	9.50 ± 0.65 ^a^	10.54 ± 1.53 ^a^	9.73 ± 1.13 ^a^	10.81 ± 2.11 ^a^

Data are mean ± standard deviation (*n* = 3). Different letters within a row indicate significant differences (*p* < 0.05). SFAs: saturated fatty acids; PUFAs: polyunsaturated fatty acids; MUFAs: monounsaturated fatty acids; EPA: C20:5n-3; DHA: C22:6n-3.

## Data Availability

The original contributions presented in the study are included in the article, further inquiries can be directed to the corresponding authors.
